# High risk of political bias in black box emotion inference models

**DOI:** 10.1038/s41598-025-86766-6

**Published:** 2025-02-19

**Authors:** Hubert Plisiecki, Paweł Lenartowicz, Maria Flakus, Artur Pokropek

**Affiliations:** 1 Institute of Psychology, Polish Academy of Sciences, Warsaw, Poland; 2 Stowarzyszenie na rzecz Otwartej Nauki (Society for Open Science), Warsaw, Poland; 3 Institute of Philosophy and Sociology, Polish Academy of Sciences, Warsaw, Poland

**Keywords:** Political Bias, Emotion inference models, Sentiment analysis, Social Science Research, Annotator Bias, Computational science, Human behaviour

## Abstract

This paper investigates the presence of political bias in emotion inference models used for sentiment analysis (SA). Machine learning models often reflect biases in their training data, impacting the validity of their outcomes. While previous research has highlighted gender and race biases, our study focuses on political bias—an underexplored, pervasive issue that can skew the interpretation of text data across many studies. We audit a Polish sentiment analysis model developed in our lab for bias. By analyzing valence predictions for names and sentences involving Polish politicians, we uncovered systematic differences influenced by political affiliations. Our findings suggest that annotations by human raters propagate political biases into the model’s predictions. To prove it, we pruned the training dataset of texts mentioning these politicians and observed a reduction in bias, though not its complete elimination. Given the significant implications of political bias in SA, our study emphasizes caution in employing these models for social science research. We recommend a critical examination of SA results and propose using lexicon-based systems as an ideologically neutral alternative. This paper underscores the necessity for ongoing scrutiny and methodological adjustments to ensure the reliability of the use of machine learning in academic and applied contexts.

**Supplementary Information:**

The online version contains supplementary material available at 10.1038/s41598-025-86766-6.

## Introduction

“*The bias I’m most nervous about is the bias of the human feedback raters.*”

~ Sam Altman, OpenAI CEO.

It is a well-documented fact that machine learning models are prone to being biased by their training data. Studies have repeatedly shown the presence of biases against various social groups, including gender and race biases, in machine learning-based sentiment analysis (SA) systems—systems that predict the positivity of text snippets^1–3^. These types of biases are significant from a social justice perspective, as they can exacerbate the reporting of spurious differences between social groups and affect the interpretation and outcomes of studies across various domains.

In this paper, we highlight another critical dimension of bias in SA systems: political bias, or the propagation of the political orientation of the annotators through the annotated data to the predictions of the SA model. Political bias has the potential to skew the interpretation of data across a wide range of studies, affecting societal perceptions and policymaking at a systemic level. Given that nearly every text contains some level of political nuance^4^, this kind of bias can potentially influence many studies that employ SA, especially in the social sciences.

The aim of this research is to show that political bias in SA systems is substantial and pervasive. This bias not only intersects with other biases reported so far, such as gender and race biases, but also extends beyond them, rendering many research conclusions less reliable. By addressing political bias, we seek to contribute to a more comprehensive understanding of biases in SA systems and to encourage the development of mitigation strategies that enhance the reliability and fairness of SA applications.

## Emotion and sentiment analysis in Social sciences

In recent years, social scientists have increasingly recognized the profound influence of emotions across a broad range of disciplines. This interdisciplinary approach has illuminated the significant role emotions play in shaping human behavior and societal dynamics in fields such as political science^5^, sociology^6,7^, economics^8^, anthropology^9^, and organizational research^10^, among others. The proliferation of text data sources—including social media, computer-based survey responses, political speeches, newspapers, online forums, customer reviews, blogs, and e-books—has provided unprecedented opportunities to examine emotions outside traditional psychological laboratory settings. Consequently, various tools have been developed to detect emotions^11,12^. As a result, research in this area has expanded rapidly.

To provide specific examples, in previous research SA been employed to predict election results^13^, gauge public sentiment toward pressing social issues^14^and compare the emotional content between news sources from different ends of the political spectrum^15^. During the COVID-19 pandemic, numerous studies analyzed public sentiment based on online data^16,17^leading to conclusions such as describing the crisis communication styles on Twitter of different Indian political leaders^18^. Similar research examined the emotional tone of the Austrian 2016 presidential election candidates^19^. From a psychological perspective, SA and emotion prediction have been used to assess suicide risk^20^, automate feedback in online cognitive behavioral therapy^21^, predict the subjective well-being of social media users^22^, and analyze the subjective well-being of people over the past centuries^23^. All of these studies relied on sentiment analysis written text to reach scientific conclusions, showcasing the importance of this technique in current social research. However, the exact implementation of SA can vary from study to study.

Overall, there exist three main categories of sentiment analysis (SA) systems: (A) dictionary-based approaches, (B) large language model (LLM) approaches, and (C) classical supervised predictive model approaches, referred to from now on as predictive model approaches for brevity. Dictionary-based approaches (A), also known as lexicon-based methods, rely on predefined lists of words associated with specific sentiments. These dictionaries, such as the AFINN, SentiWordNet, and LIWC^24–26^, assign sentiment scores to words and phrases within a text to determine its overall sentiment. This method is straightforward and interpretable, but it can be limited by the coverage and accuracy of the dictionary, as well as by the inability to capture contextual information.

In contrast, large language model (LLM) approaches (B) leverage advanced neural networks trained on vast amounts of text data. Models such as GPT-4, LLAMA, and their derivatives can capture nuanced sentiment by understanding the context and relationships between words in a sentence and predict it in a zero-shot (without any examples to guide it), or multiple shot manner (with examples). However, their performance in emotion detection specifically falls short of the state-of-the-art (SOTA) predictive model approaches (C)^27^.

The predictive model approach (C) involves training machine learning-based classifiers or regressions on labeled datasets. Techniques such as support vector machines, random forests, and deep learning models are used to predict sentiment based on features extracted from the text. These approaches are currently considered the best for analyzing emotion according to robust tests of prediction accuracy on political text datasets, as well as broader domain benchmarks^27,28^. However, this high accuracy comes at the cost of lower interpretability and, as this study will underline, a propensity for bias.

## Bias in predictive models

Bias in predictive models originates from the training data, which in the case of sentiment analysis (SA), consists of annotated text datasets. These datasets are the result of the laborious work of annotators who read through provided materials and assign emotional labels. Annotators can differ on many accounts, including age, gender, socio-economic status, psychological individual differences, and political orientation. All these differences can impact the annotation process. Studies such as Milkowski and associates^29^have shown that individual differences among annotators can significantly affect emotion annotations in text. These individual differences introduce subjectivity into data assumed to be objective, leading to inconsistencies that can skew the training and evaluation of models designed to predict emotional reactions from text. Moreover, annotation bias can result from a mismatch between authors’ and annotators’ linguistic and social norms, as noted by Sap and colleagues^30^. This mismatch often reflects broader social and demographic differences that can manifest in critical research areas like hate speech and abuse detection. For instance, studies by Larimore and associates^31^, and Waseem^32^ show that the race and gender of annotators influence not only the annotation process but also the performance of NLP models, further compounding biases.

Particularly concerning is the influence of annotators’ political and ideological biases. This type of bias not only includes biases against specific social groups reported in earlier studies, but its generality makes the specific extent of its influence on SA models difficult to determine, although we expect it to be significant^1–3^. Ennser-Jedenastik and Meyer^33^report that coders of political texts often incorporate their prior beliefs about political parties into their coding decisions. For example, annotators are more likely to perceive a sentence as supporting immigration if they believe it comes from a left-wing party, regardless of the actual content. Experimental studies by van der Velden^34^show that personal characteristics of annotators, like political ideology or knowledge, interfere with their judgment of political stances. It’s important to note that this interference might not be fully realized by the annotator, as previous psychological studies have shown the influence of political orientation on implicit judgments^35,36^. Here of significant importance are the findings that show that people of different political orientations differ significantly in many annotation tasks related to political science, including emotion annotation of images^37^. This means that constructing an annotation strategy that eliminates the propagation of individual bias to SA models might be problematic. This problem parallels many similar ones in algorithm creation, where the human behavior information, on which the model is trained, falls short of the aim of the engineered algorithm. In such cases, Morewedge and associates^38^ recommend auditing the models under suspicion by testing them for the presence of bias directly.

## Current study

In this study, we conduct a bias audit of an existing Polish sentiment analysis model developed by our lab as a part of a different research endeavor^39^ to determine whether its predicted valence ratings show systematic differences based on the party affiliation of a diverse group of politicians from different political parties. We predict the valence of the names of the politicians, as well as sentences in which their names are embedded to vary based on their political affiliation (the latter were included to analyze both the direct valence towards the politicians as well as take into account the usual settings in which such a model would be used, where the name of the politicians would be a part of a specific sentence.) We regress the political affiliation of the politicians onto the sentiment readings of the model to see how much variance it can explain. To pinpoint the source of the bias, we prune the training set of any mentions of the aforementioned politicians, train a second model, and repeat the analysis.

## Results

### Regression models

The predictive model returns the valence metric as a continuous score scaled to a range from 0 to 100. When applied to the 24 names of politicians selected for analysis, the valence scores ranged from 42.3 to 56.6, with an average (not weighted) (M) of 49.5 and a standard deviation (not weighted) (SD) of 3.17. To examine potential bias in more natural contexts, we estimated valence for names embedded in both neutral and politically charged sentences. The mean valence was higher in neutral sentences (M = 54.4) compared to raw names (M = 49.5) and lower in politically charged sentences (M = 45.7). Interestingly, the differences in valence among politicians (measured by the standard deviation of valence) were larger for neutral sentences (SD = 4.35) compared to raw names (SD = 3.17), and smaller for politically charged sentences (SD = 1.29).

**Fig. 1:**
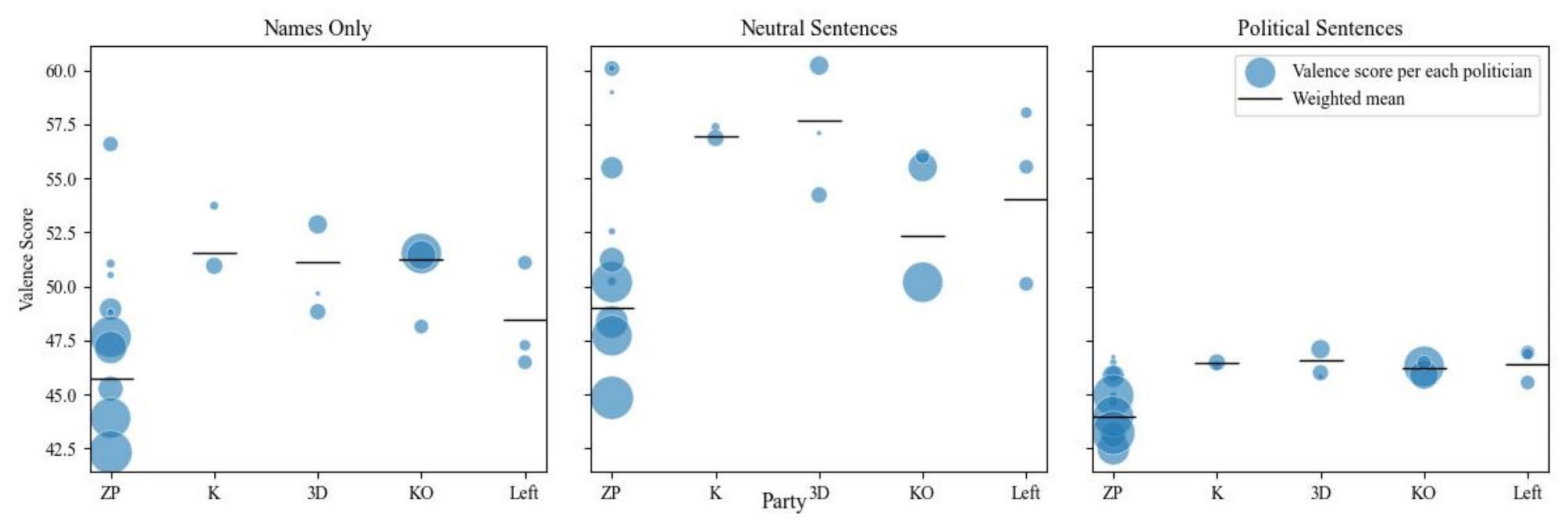
Predicted valence scores of the names of the politicians. Note: the area of the dots corresponds to the weight (the amount of tweets containing the name of a politician). Abbreviations: ZP – Zjednoczona Prawica, K – Konfederacja, 3D – Trzecia Droga, KO – Koalicja Obywatelska, Left – Nowa Lewica.

Along with the visualization of the differences in predicted valence scores of politicians’ names (See Fig. 1), we regressed these valence scores, as well as the predicted valence scores for the aforementioned sentences, onto the independent variables of interest (Table 1). The fitted models with politicians’ affiliation and gender (the reasoning for the inclusion of the gender confounder is driven by analyses explained in the later section Confounds) seem to describe the data well, and explain 66.5%, 52% and 66.2% of variance (R²). All of the coefficients have the same direction and similar magnitudes in all of the three models (Model 1, 2, and 3). The hypothesis of exchangeability of scores^55^ could be rejected due to low p-values: *p* = 0.008, *p* = 0.049 and *p* = 0.018, which implies that the differences in valence are not random.

**Table 1:** Regression models – differences in valence.

	Dependent variable: Valence of:		
	Names only	Neutral Sentences	Political Sentences
	(1)	(2)	(3)
intercept	45.40***	48.61***	43.89***
	(0.67)	(0.92)	(0.26)
3D	5.73**	9.09**	2.72**
	(2.37)	(3.26)	(0.93)
K	6.15*	8.37*	2.56*
	(3.1)	(4.28)	(1.22)
KO	5.83***	3.71*	2.30**
	(1.31)	(1.8)	(0.51)
Left	3.03	5.46	2.51**
	(2.56)	(3.53)	(1.01)
gender	9.77**	10.10*	1.88
	(3.48)	(4.8)	(1.37)
Observations	22	22	22
R²	0.665	0.52	0.662
Adjusted R²	0.547	0.37	0.556
Residual Std. Error	3.38 (df = 16)	5.21 (df = 16)	1.29 (df = 16)
P-value (permutation)	0.008	0.049	0.018

Note: *p < 0.1; **p < 0.05; ***p < 0.01 (*t-*test).

Zjednoczona Prawica (ruling party) as intercept, gender: woman = 1, man = 0.

K – Konfederacja, 3D – Trzecia Droga, KO – Koalicja Obywatelska, Left – Nowa Lewica.

### Confounds

The association of political affiliation and valence was significantly stronger than between valence and the confounders. This is evidenced by comparing R² of regression on valence in raw names (Model 1, Table 2.) and political affiliation (R² = 0.49), and models with only confounds as independent variables. The model including only gender reared R² = 0.109 (statistics that relate to gender should be interpreted carefully since there are only 2 women in our sample), trust towards Zpolitician achieved R² = 0.195, and the mean valence of mentions in which a given politician appeared resulted in R² = 0.175. (These models are available in the Appendix)

To find the model that best describes the data, we compare adjusted R² with different sets of potential confounds. Since the model with affiliation and gender as independent variables has the highest adjusted R², this set of independent variables is used in other models. (See Table 2.)

**Table 2:** Regression models – inspecting confounders.

	Dependent variable: Valence in raw names				
	(1)	(2)	(3)	(4)	(5)
intercept	45.76***	45.40***	45.52***	45.76***	46.06***
	(0.78)	(0.67)	(0.69)	(1.38)	(1.43)
3D	5.36*	5.73**	5.55**	5.14	4.67
	(2.8)	(2.37)	(2.39)	(3.14)	(3.19)
K	5.79	6.15*	5.86*	4.91	4.01
	(3.67)	(3.1)	(3.14)	(5.23)	(5.35)
KO	5.47***	5.83***	5.40***	5.69***	5.16***
	(1.54)	(1.31)	(1.41)	(1.43)	(1.55)
Left	2.67	3.03	3.01	2.71	2.54
	(3.03)	(2.56)	(2.86)	(2.85)	(2.87)
gender		9.77**	8.72*	9.61**	8.40**
		(3.48)	(3.71)	(3.63)	(3.88)
trust			0.71		0.76
			(0.8)		(0.83)
mentions				0.037	0.055
				(0.124)	(0.127)
Observations	22	22	22	22	22
R²	0.485	0.655	0.672	0.657	0.676
Adjusted R²	0.364	0.547	0.541	0.52	0.514
Residual Std. Error	3.88	3.38	3.39	3.35	3.31
	(df = 17)	(df = 16)	(df = 15)	(df = 15)	(df = 14)
P-value (permutation)	0.051	0.008	0.021	0.017	0.039

*p < 0.1; **p < 0.05; ***p < 0.01 (t-test).

Zjednoczona Prawica (ruling party) as intercept, gender: woman = 1, man = 0, trust: normalized trust scores, mentions: mean valence of annotated text, in which politician was mentions in 0–100 scale.

K – Konfederacja, 3D – Trzecia Droga, KO – Koalicja Obywatelska, Left – Nowa Lewica.

### The modified model

In the model modified (See Table 3.) by pruning texts containing mentions of our set of politicians, the relationships between affiliation and valence decreased significantly, but bias was still present in the model with raw names (Model 1., Table 3.) It should be noted that not all mentions affecting the model could be pruned, for example, the most mentioned politician is Jarosław Kaczyński, but in the dataset there are tweets mentioning his twin brother, Lech Kaczyński, the former president and member of the same party.

**Table 3:** Regression models – modified model (text pruning).

	Dependent variable: Valence of: (modified model)		
	raw names	neutral sentences	political sentences
	(1)	(2)	(3)
intercept	49.48***	53.85***	45.09***
	(0.51)	(1.14)	(0.34)
3D	3.70*	5.93	0.77
	(1.79)	(4.00)	(1.20)
K	2.69	4.64	0.41
	(2.35)	(5.26)	(1.58)
KO	1.55	0.87	0.75
	(0.99)	(2.21)	(0.66)
Left	0.76	3.38	0.9
	(1.94)	(4.34)	(1.30)
gender	6.47**	7.74	0.77
	(2.63)	(5.90)	(1.77)
Observations	22	22	22
R²	0.421	0.224	0.104
Adjusted R²	0.24	−0.019	−0.176
Residual Std. Error	3.02 (df = 16)	4.87 (df = 16)	1.10 (df = 16)
P-value (permutation)	0.076	0.101	0.202

*p < 0.1; **p < 0.05; ***p < 0.01 (t-test).

Zjednoczona Prawica (ruling party) as intercept, gender: woman = 1, man = 0.

K – Konfederacja, 3D – Trzecia Droga, KO – Koalicja Obywatelska, Left – Nowa Lewica.

## Discussion

In the current study we have shown that a supervised model trained on annotations created by expert annotators in their domain shows signs of political bias with regards to well-known politicians. The impact of this bias depends on the analytical context. While using our original model, introducing a politically charged surname can alter the sentiment score of single text snippet by up to 6 points on a 1–100 scale or by 0.5 in terms of Cohen’s d. Given the effect size, datasets with minimal politically charged content may experience only minor bias issues. However, when comparing political groups—as in regression analyses across different political parties—this bias becomes systematic. For instance, the difference between groups, when compared to the pooled standard deviation, is 5.47 versus 3.53 (Cohen’s d = 1.55) for raw names, or 2.30 versus 1.23 (Cohen’s d = 1.87) for modified names. This suggests that bias could be even more pervasive in datasets with greater political content and in inter-group analyses.

Interestingly, while the variance in sentiment predictions of raw names, and neutral sentences was higher than in the case of political sentences, which most probably relates to the higher level of emotional signal present in the latter as the words in political sentences contained more emotionally charged language such as “support”, or “against”, the systematicity of the bias present in political sentences counteracted this effect as the regression models conducted on each of these conditions explained similar levels of variance (0.665, 0.52, and 0.662 consecutively). This may suggest that the presence of additional political context in the predicted text could prime the model to focus on the political information, thereby making the bias more systematic.

Of note is the fact that this bias was not explained by the publics’ trust towards politicians, indicating that it does not reflect the general society’s political preferences, but rather those of a selected nonrepresentative group. Similarly, the mean valence of the training data tweets that included the names of the politicians did not explain this bias either. This rules out the hypothesis that the bias comes from a systematic difference in valence between how the politicians were portrayed in text, or other text-inherent reasons. Another argument against the influence of the linguistic bias is the moderate intraclass correlation coefficient (0.6) indicating limited agreement between the annotators which further undermines the possibility of the bias being inherent to the text of the training dataset, and not to the subjective perception of the text by the annotators.

The modified model, trained on a dataset pruned of texts containing politicians’ names, exhibited significantly lower bias than the primary model suggesting that at least a substantial part of the bias can be attributed to the annotations made by the annotation team in a causal manner. It, however, does not indicate that pruning the names of the politicians eradicates all kinds of biases that political orientation might result in. Additionally, the model cannot be fully isolated from the influence of certain mentions that may affect its output. For instance, while identifying mentions of Jarosław Kaczyński, snippets related to his twin brother, Lech Kaczyński, might be included, potentially influencing the model’s predictions. More indirect sources of bias might also be present. Moreover, certain word associations may be embedded in the model’s initial architecture before training for emotion detection, and this pre-existing knowledge could interact with the annotations producing harder to eradicate bias. Given these limitations, along with broader challenges in practical application, we do not recommend pruning as a method for bias mitigation. Furthermore, the instructions given to the annotators, which prompted them to estimate the “positivity/negativity that they read in each text” rather than their emotional reactions to it, leads us to the conclusion that the bias propagated into the annotated dataset in an implicit manner. Instances of such implicit propagation of political orientation have been documented in previous psychological research^35,36^.

The most likely explanation for this effect is that when annotators saw a text mentioning a politician, they tended to label it in accordance to their own political orientation. During training, these biased labels were treated as ground truth. Consequently, the model learned to attribute any difference in valence between a text containing the politician’s name and a similar text without it to the politician’s name itself. Repeated exposure to this pattern reinforced a systematic bias. By removing texts containing such biased annotations, we therefore reduced this bias.

Because the model was originally created for a separate study, we lack detailed information about each annotator’s political orientation, making it impossible to directly correlate their political views with the observed bias. Nonetheless, we contacted the annotators post hoc and invited them to complete an anonymous, voluntary survey on their political orientation, to which 15 out of the 20 responded. The results, while generally consistent with the observed bias, offer only tentative evidence for its propagation, and are therefore presented in the appendix.

The existence of political bias in the model has been clearly documented, and so has its causal link to the training data. Direct evidence that this bias aligns precisely with the political orientations of individual annotators is limited, as we lack complete information about their political preferences. However, this limitation does not weaken the conclusion that the model’s bias was learned from the annotation process. First, the bias does not reflect society-wide patterns of trust toward these politicians. Second, pruning data that mentioned political figures significantly reduced the bias, supporting the idea that the skew originated in annotations. Finally, a post-hoc, voluntary survey of annotators—albeit incomplete—revealed trends consistent with the observed bias.

These findings highlight that annotator-based biases can readily transfer to trained models, even when instructions direct annotators to judge the text rather than their personal feelings about it. Although the post-hoc survey provides only preliminary insight into how annotators’ political leanings might have shaped their labels, such information is not strictly necessary to conclude that the model is biased. The most plausible interpretation remains that model bias stems from the subjective political perceptions of a subset of annotators, whose labeling patterns the model then learned. This means not only that the annotations made by humans can lead to biased models, but also raises the very real possibility that their bias might have spread to more concepts in the dataset. If people implicitly propagate their political orientation towards social groups^1–3^ as well as specific politicians as proven by the current study, the only thing standing in the way of abstract concepts being affected by the same type of bias is the ability of the model to pick up on it.

As language models become more advanced, their understanding of language becomes gradually less reliant on specific entities which they pick up from the text as in the case of for example Naive Bayes algorithms^40^, and more reliant on relations between abstract concepts. This is evidenced by the distributed nature of the information that large language models and other transformer-reliant architectures use, through the mechanisms of attention, to generate their outputs^41^, as well as by the recent LLM interpretability research showcasing the crystallization of abstract concepts within the inner layers of these models^42^. This means that as models improve, the propensity of the models being biased towards specific abstract concepts such as for example anarchism, or democracy might increase, given that such bias will be present in the training data, which is likely. Furthermore, the inspection of these kinds of distributed, conceptual biases will require new, more complex methods of bias detection.

Given the biases that have been already uncovered in SA models, as well as those more abstract that can lurk in the shadows, yet unidentified, we discourage the use of such models for research and advise caution in interpreting the results of those that have already used them. To stress the kinds of problems their use can lead to, let’s go back to the examples of research performed with the use of SA systems. The analysis of the sentiment towards social issues might be biased towards the sentiment of the annotator’s team^14^. Similarly, when comparing emotional content of news sources, the same propagation of bias can occur^15^, directly biasing the conclusions. This problem of propagation of bias directly biases studies that apply their SA systems to compare different groups of texts in terms of emotionality. When trying to predict something using SA scores, like in the case of predicting election results, assessing suicide risk, or subjective wellbeing, the effectiveness of the predictive model can be influenced by the beliefs of the annotator group, leading to replication issues^13,20,22^. At the same time, when creating customer facing solutions such as automating feedback in online cognitive behavioral therapy one has to consider that annotator biases might lead to people with different political predispositions receiving different standards of care, however here the influence is not as clear cut as in earlier cases.

However, some of the studies mentioned in this paper may be less affected by this bias, as many of them have relied on lexicon-based SA systems, forgoing the increased accuracy of the predictive models in exchange for elevated transparency. As these approaches depend on lists of emotionally loaded words which are not ideologically relevant, annotated separately, and without any contexts, they are significantly less susceptible to propagating the bias of their annotators. Furthermore, any bias that they do propagate can be clearly read from the word annotations themselves, therefore researchers that want to buttress their analysis against specific biases can directly check for them within the lexicon and correct them there and then. The same task is orders of magnitude more complicated when using black box ML models and becomes even more complicated when the bias concerns concepts rather than entities. Lexicons, however, should not by any means be assumed to be bias-free, but rather less susceptible to carry it, and easier to buttress against it.

The higher accuracy of transformer-based, and other predictive models could be therefore traded in for the less accurate, but more bias- safe lexicon-based systems. However, given that the drop in performance when using lexicon approaches is quite severe this might not be a preferred solution for some researchers^28^. Additionally, lexicons might exhibit different types of biases – such as those related to lists of words that are not representative of their natural language use. They should, therefore, also be used with caution. Future research should therefore focus on creating emotion prediction ML models that are more robust to training bias. In the case where authors choose to use ML based SA systems anyway, we recommend them to take the possibility of different types of potential biases into consideration when analyzing their results, and if possible, to corroborate their results using a lexicon-based system.

The alternative in the form of picking the annotation team so that it is balanced with regards to all of the individual differences such as political orientation and others that could influence their annotations is problematic as (1) it is as of yet not clear which differences could play a role in the annotation process (2) balancing a large number of them would require a very large annotation team which would be very resource intensive. Nonetheless in very specific applications where the nature of the bias relevant to a given experiment can be directly pinpointed such solutions might be viable.

In conclusion, the current paper shows that supervised models trained on datasets annotated by humans are susceptible to showing the same biases as annotators, despite the annotation instructions being phrased in a way that should avoid the propagation of such bias. This result should be taken into consideration when conducting and interpreting sentiment analysis research in the political science sphere and beyond. We therefore recommend the research community to perceive machine learning based sentiment analysis models as biased until proven otherwise and consider exploring alternative approaches.

The main limitation of the current study is its focus on a single sentiment analysis model and a specific dataset largely composed of political texts in Polish. While these conditions are ideal for exploring political bias within the context of Polish politics, the generalizability of the findings cannot be stated with certainty, although should be taken into consideration. We recommend the researchers that are in doubt about whether our results extend to their models to replicate our findings before using them. Additionally, the sample size of politicians and the specific sentences used to assess bias were relatively small, which may limit the robustness of our regression analyses. Future research should aim to replicate these findings across diverse datasets, expand the number of annotators and the range of their political orientations, and explore the interaction between different types of bias in sentiment analysis models. Explorations of the exact mechanisms through which the bias is propagated would also be insightful from a psychological perspective, and perhaps could bolster the development of bias-safe emotion prediction alternatives.

## Methods

### The prediction model

#### Model training data

The model has been trained on a training set sampled from a comprehensive database of Polish political texts from social media profiles (i.e., YouTube, Twitter, Facebook) of 25 journalists, 25 politicians, and 19 non-governmental organizations (NGOs). The complete list of the profiles is available in the Appendix. For each profile, all available posts from each platform were scraped (going back to the beginning of 2019). In addition, we also included texts written by “typical” social media users, i.e., non-professional commentators of social affairs. Our data consists of 1,246,337 text snippets (Twitter: 789490 tweets; YouTube: 42252 comments; Facebook: 414595 posts).

As transformer models have certain limits, i.e., their use imposes limits on length, we implemented two types of modification within the initial dataset. First, since texts retrieved from Facebook were longer than the others, we have split them into sentences. Second, we deleted all texts that were longer than 280 characters.

The texts were further cleaned from social media artifacts, such as dates scrapped alongside the texts. Next, the *langdetect*^43^ software was used to filter out text snippets that were not written in Polish. Also, all online links and usernames in the texts were replaced with “_link_” and “_user_”, respectively, so that the model does not overfit the sources of information nor specific social media users.

Because most texts in the initial dataset were emotionally neutral, we filtered out the neutral texts and included only those that had higher emotional content in the final dataset. To filter the neutral snippets, the texts were stemmed and subjected to a lexicon analysis^44^ using lexical norms for valence, arousal, and dominance - the three basic components of emotions. The words in each text were summed up in terms of their emotional content extracted from the lexical database and averaged to create separate metrics for the three emotional dimensions. These metrics were then summed up and used as weights to choose 10,000 most emotionally loaded texts for the final training dataset. The proportions of the texts coming from different social media platforms reflected the initial proportions of these texts, resulting in 496 YouTube texts, 6105 Twitter texts, and 3399 Facebook texts, totaling 10,000 texts.

#### Annotators

The final dataset consisting of 10,000 texts was annotated by 20 expert annotators (age: M = 23.89, SD = 4.10; gender: 80% female) with regards to six emotions: happiness, sadness, disgust, fear, anger, and pride, as well as to two-dimensional emotional metrics of valence and arousal, using a 5-point Likert scale. All annotators were well-versed in Polish political discourse and were students of Psychology (70% of them were graduate students, which in the case of Polish academic education denotes people studying 4th and 5th year). Thus, they underwent at least elementary training in psychology. Each text was annotated by 5 randomly picked annotators. The inter annotator reliability as measured by the intraclass correlation coefficient (ICC(1)) for valence measured 0.60 indicating moderate reliability^45^.

Since valence and arousal might not have been familiar to annotators, before the formal annotation process began, all annotators were informed about the characteristics of valence and arousal. General annotation guidelines were provided to ensure consistency and minimize subjectivity. For the purpose of annotating valence of texts, the annotators were given the following instruction:

English translation (An in-depth description of the annotation process is available in the Appendix):

Go back to the text you just read. Now think about the sign of emotion (positive / negative) and the arousal you read in a given text (no arousal / extreme arousal). Rate the text on these emotional dimensions.

#### Model training

For model training, we have considered two alternative base models: the Trelbert transformer model developed by a team at DeepSense^46^, and the Polish Roberta model^47^. The encoders of both models were each equipped with an additional regression layer with a sigmoid activation function. The models have been trained to predict each of the six emotion intensities, as well as valence, and arousal. The maximum number of epochs in each training run was set to 100. At each step, we computed the mean correlation of the predicted metrics with their actual values on the evaluation batch, and the models with the highest correlations on the evaluation batch were saved to avoid overfitting. We used the MSE criterion to compute the loss alongside the AdamW optimizer with default hyperparameter values. Both of the base models were then subjected to a Bayesian grid search using the WandB platform^48^ with the following values: dropout − 0; 0.2, 0.4, 0.6; learning rate − 5e-3, 5e-4, 5e-5; weight decay − 0.1, 0.3, 0.5; warmup steps − 300, 600, 900. The model which obtained the highest correlation relied on the Roberta transformer model and had the following hyperparameters: dropout = 0.6; learning rate = 5e-5; weight_decay = 0.3. Its average accuracy on the test set is *r* = 0.80, and *r* = 0.87 valence, which is the main metric analyzed in the current study as it shows the estimated general positivity of the analyzed text.

### Bias testing

#### Stimuli

As stimuli for testing the bias hypothesis, to limit our arbitrary choice of stimuli, we used the names of 24 well-known Polish politicians who appeared in the November and October 2023 trust polls^49–51^. The politicians were assigned to 5 political parties/coalitions on the basis of their affiliation or because they were candidates of that party/coalition. These parties/coalitions are Zjednoczona Prawica, which is right-wing and was the ruling coalition, Trzecia Droga, Koalicja Obywatelska, Nowa Lewica, which were centre-right, centre and left opposition respectively. The fifth party was Konfederacja, which was a right to far right opposition. These coalitions cover 96.25% of the total votes in the November 2023 parliamentary elections^52^.

The model was used to predict the valence for each of the aforementioned stimuli. While capable of estimating the intensity of other affective metrics, the choice of valence is both natural and self-evident: valence, by definition, reflects the positive or negative reaction to a stimulus. No other affective metric aligns as directly with the binary essence of approve/disapprove evaluations, making valence the most intuitive and robust indicator of bias in politically charged contexts. To further examine this, we predicted the valence of politicians’ names in isolation, as well as in neutral and politically contextualized sentences, to estimate how their inclusion alters the model’s predictions. Details of these stimuli are provided in the appendix.

#### Corpus modification

To identify the potential source of the model’s bias, we locate the texts in the training set that contain the surnames of these politicians. We then manually review these texts to see if they are referring to a particular politician. There are 459 of these texts in total, with a range of 71 to 0 and a median of 8.5 per politician. We then prune the training set of these texts and train a second model with the same training parameters to estimate the degree to which their presence influences the model’s bias. The training set contained 7999 texts before pruning, which means that the pruned texts constitute below 6% of its size.

### Statistical analyses

To test for the presence of bias, we examine where there are noticeable differences in the valence of politicians’ names and where they can be explained by the politicians’ political affiliations. For this purpose, we build several regression models. As dependent variables, we use the valence score from the original model, the same score from the modified model (trained on the pruned corpus), and the differences in valence between the final and the modified model. The models return the valence score as continuous variables ranging from 0 to 1, which we chose to then recalculate on a 0–100 scale for better readability.

As independent variables we use the politicians’ affiliation, and potential confounders: their gender, trust towards them (from the same trust surveys as the names of politicians) and mean annotated valence of texts in which these politicians appear, recalculated to 0–100. We included a trust “score” as a proxy to control for the general favourability of each politician, to separate it from nonrepresentative political bias. This allows us to account for positive or negative feelings about a politician that may stem from their popularity or personal traits rather than political alignment. Additionally, the mean valence of the training dataset snippets with the politicians’ names was included to test the possibility that the bias of the model stems from text-inherent sources such as biased language. This could mean for example that certain politicians were accompanied by more negative language than others, translating to biased training. By incorporating both trust and mean annotated valence scores, we aimed to rule out alternative explanations for differences in valence beyond the annotators’ political bias.

The trust surveys were decoded as 5-point Likert scales^51^or 3-point Likert scales^49,50^. Responses “I don’t know” and “difficult to say” were recoded as neutral. For each survey, a normalized score was calculated, and the mean of these normalized scores was included in the analysis. Mean annotated valence scores were derived from texts that were later pruned, see ‘Corpus Modification’, and recalculated to 0–100 scale.

For the regression models we use the weighted least squares method^53^, weighted by the number of mentions of a given politician. Due to the weighting process, two politicians without any mentions in training data were excluded. To test the null hypothesis of lack of correlation between political affiliation and bias in the models, we conducted the permutation tests on the observed valence (Manly, 1997) for each model, with 100,000 random assignments. This method guarantees robustness and decent statistical power^54^. This method could be vulnerable to extreme outlier in dependent variables, which is not a problem in this study, due to the categorical or bounded character of dependent variables used in this study. The QQ-plots of the model residuals are included in the appendix. Due to small sample sizes for affiliations, parametric (assuming normal distributions) confidence intervals are calculated.

## Electronic supplementary material

Below is the link to the electronic supplementary material.

Supplementary Material 1

## Data Availability

The code and data used in the current study is available at the github repository https://github.com/hplisiecki/political-model-bias and the https://osf.io/q8bes/?view_only=6f246610bc0b43cc9e98d7c978f2f6fa . The base model used for the current study is available at https://huggingface.co/hplisiecki/polemo_intensity , while the modified model is available at the aforementioned OSF repository.
