# Publisher Correction: High risk of political bias in black box emotion inference models

**DOI:** 10.1038/s41598-025-97340-5

**Published:** 2025-04-11

**Authors:** Hubert Plisiecki, Paweł Lenartowicz, Maria Flakus, Artur Pokropek

**Affiliations:** 1 Institute of Psychology, Polish Academy of Sciences, Warsaw, Poland; 2 Stowarzyszenie na rzecz Otwartej Nauki (Society for Open Science), Warsaw, Poland; 3 Institute of Philosophy and Sociology, Polish Academy of Sciences, Warsaw, Poland

Correction to: *Scientific Reports* 10.1038/s41598-025-86766-6, published online 19 February 2025

The Funding section in the original version of this Article was omitted.

The Funding section now reads: “This research is funded by a grant from the National Science Centre (NCN) ‘Research Laboratory for Digital Social Sciences’ (SONATA BIS-10, No. UMO-020/38/E/HS6/00302).”

The original Article has been corrected.

