# Supplementary material for: High risk of political bias in black box emotion inference models
Source: Sci Rep. 2025 Feb 19;15:6028. doi: 10.1038/s41598-025-86766-6 (PMC11840103; doi:10.1038/s41598-025-86766-6)
Supplement: Supplementary file 1 — Supplementary Material 1 [file 41598_2025_86766_MOESM1_ESM.docx]

**1. Annotation Process**

The final dataset consisting of 10,000 texts was annotated by 20 expert annotators (age: M = 23.89, SD = 4.10; gender: 80% female). All annotators were well-versed in Polish political discourse and were students of Psychology (70% of them were graduate students, which in the case of Polish academic education denotes people studying 4th and 5th year). Thus, they underwent at least elementary training in psychology.

The entire annotation process lasted five weeks. Each week, every annotator was given five sets of texts (out of 100 sets with 100 randomly assigned sentences each) that should be annotated in the given week. The sets were randomly assigned to annotators, taking into account the general assumption that five different annotators should annotate each set. Generally, annotators simultaneously annotated no more than 500 texts each week, preventing them from cognitive depletion's negative effects.

Annotators labeled each text based on five basic emotions: happiness, sadness, anger, disgust, and fear. In addition, annotators were asked to label the texts with regard to additional emotion, namely pride, and two general dimensions of emotions: valence and arousal. In all cases, annotators used a 5-point scale (in the case of emotions: 0 = *emotion is absent*, 4 = *very high level of emotion*; in the case of valence and arousal, we used a pictographic 5-point scale provided in Supplementary materials).

Since two additional emotional dimensions might not have been familiar to annotators, before the formal annotation process began, all annotators were informed about the characteristics of valence and arousal (note that we did not provide formal definitions of basic emotions). General annotation guidelines were provided to ensure consistency and minimize subjectivity.

**2. Social Media Profiles**

The Social Media profiles scraped to generate the training dataset:

1. Journalists:

Adrian Klarenbach, Agnieszka Gozdyra, Bartosz T. Wieliński, Bartosz Węglarczyk, Bianka Mikołajewska, Cezary Krysztopa, Daniel Liszkiewicz, Dawid Wildstein, Dominika Długosz, Dominika Wielowieyska, Ewa Siedlecka, Jacek Karnowski, Jacek Kurski, Jacek Nizinkiewicz, Janusz Schwertner, Jarosław Olechowski, Konrad Piasecki, Krzysztof Ziemiec, Łukasz Bok, Łukasz Warzecha, Magdalena Ogórek, Magdalena Rigamonti, Marcin Gutowski, Marcin Wolski, Michał Karnowski, Michał Kolanko, Michał Rachoń, Miłosz Kłeczek, Paweł Żuchowski, Piotr Kraśko, Piotr Semka, Radomir Wit, Rafał Ziemkiewicz, Renata Grochal, Robert Mazurek, Samuel Pereira, Szymon Jadczak, Tomasz Lis, Tomasz Sakiewicz, Tomasz Sekielski, Tomasz Sommer, Tomasz Terlikowski, Wojciech Bojanowski, Agaton Koziński, Piotr Witwicki, Jacek Tacik, Magdalena Lucyan, Agata Adamek, Kamil Dziubka, Jarosław Kurski, Dorota Kania, Ewa Bugala, Zuzanna Dąbrowska, Karol Gac, Marcin Tulicki, Marzena Nykiel, Jacek Prusinowski, Paweł Wroński

1. Politicians:

Donald Tusk, Andrzej Duda, Rafał Trzaskowski, Mateusz Morawiecki, Sławomir Mentzen, Janusz Korwin-Mikke, Grzegorz Braun, Szymon Hołownia, Radosław Sikorski, Krzysztof Bosak, Władysław Kosiniak-Kamysz, Borys Budka, Artur E. Dziambor, Marek Belka, Leszek Miller, Mariusz Błaszczak, Roman Giertych, Franek Sterczewski, Konrad Berkowicz, Marek Jakubiak, Michał Szczerba, Przemysław Czarnek, Zbigniew Ziobro, Krzysztof Brejza, Leszek Balcerowicz, Izabela Leszczyna, Klaudia Jachira, Janusz Piechociński, Patryk Jaki, Robert Biedroń, Krystyna Pawłowicz, Katarzyna Lubnauer, Anna Maria Sierakowska, Łukasz Kohut, Marcin Kierwiński, Anna Maria Żukowska, Marian Banaś, Dariusz Joński, Kamila Gasiuk-Pihowicz, Barbara Nowacka, Adrian Zandberg, Krzysztof Śmieszek, Paulina Matysiak, Paweł Kukiz, Michał Wójcik, Sebastian Kaleta, Małgorzata Wassermann, Joachim Brudziński, Maciej Konieczny, Marcelina Zawisza

1. NGOs:

Polska Akcja Humanitarna, Helsińska Fundacja Praw Człowieka, Polski Czerwony Krzyż, Fundacja Dialog, Fundacja Ocalenie, Fundacja Ogólnopolski Strajk Kobiet, Stowarzyszenie Amnesty International, Fundacja Centrum Praw Kobiet, Stowarzyszenie Sędziów Polskich IUSTITIA, Stowarzyszenie Marsz Niepodległości, Lekarze bez Granic, Fundacja TVN, Fundacja Dzieciom "Zdążyć z Pomocą", Wielka Orkiestra Świątecznej Pomocy, Szlachetna Paczka, Fundacja WWF Polska, Fundacja Greenpeace Polska, Liga Ochrony Przyrody, Związek Stowarzyszeń Polska Zielona Sieć, Młodzieżowy Strajk Klimatyczny, Stowarzyszenie Miłość Nie Wyklucza, Kampania Przeciw Homofobii, Stowarzyszenie Lambda - Warszawa, Fundacja Trans-Fuzja, Stowarzyszenie Grupa Stonewall.

**3. Instructions for annotators**

You will evaluate the emotional content displayed in some short texts.

Your task will be to mark on a five-point scale the degree to which you think that a given sentence is characterized by each of the following emotions: joy, sadness, anger, disgust, fear, and pride. Use a five-point scale as described below:

0 - the emotion does not occur at all

1 - low level of emotion

2 - moderate level of emotion

3 - high level of emotion

4 - very high level of emotion.

Then, we will ask you to estimate the intensity of two additional emotion parameters: the direction of sensations (negative *versus* positive) and emotional arousal (no arousal *versus* extreme arousal). On the next screen you will learn the definitions of both parameters and how you will evaluate them.

Read the descriptions of two emotion parameters: the sign of sensations and emotional arousal. You can do this several times to make sure you understand them - it will make it easier for you to complete the task ahead of you.

You will rate each of the emotion dimensions described above on a five-point scale. To make it easier to imagine the states we have in mind, you can use pictograms symbolizing different directions of experiences and the intensity of the emotional states.

For the direction of sensations, use the following scale:

Figure S1.

*Valence response scale*


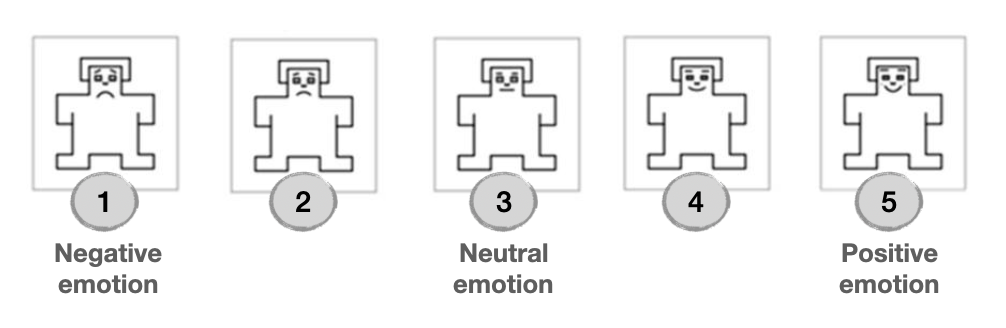


The first pictogram shows a person who is visibly depressed - specific experiences may include: panic, irritation, disgust, despair, failure, or crisis. The last image shows a person who is visibly excited - specific experiences may include: fun, delight, happiness, relaxation, satisfaction, or rest. The remaining pictograms represent intermediate states.

For emotional arousal, use the following scale:

Figure S2.

*Arousal response scale*


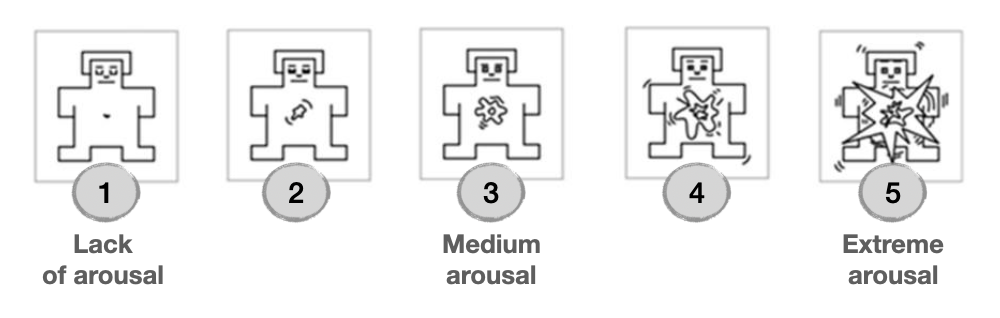


The first pictogram shows a person who is very calm, almost sleepy - specific experiences may include: relaxation, calm, inactivity, meditation, boredom, or laziness. The last image shows a person who is intensely aroused - appropriate emotional states may include: excitement, euphoria, arousal, rage, agitation, or anger.

Save the link to this manual for later - you can return to it at any time during the examination.

Very important: you can take a break while assessing your statements and return to them at any time - your current work will be saved and you will be able to resume it after the break. If you want to do this, in the upper right corner of the screen you will find the option: "Postpone for later" - click on it, enter the data necessary to save, and confirm the operation. In case you are ready to get back to work: when you enter the study page, an option "Load unfinished survey" will appear in the upper right corner of the screen - select it to load your work.

**4. Politicians’ names, Neutral and Political Sentences**

Politicians’ names:

Władysław Kosiniak-Kamysz, Szymon Hołownia, Andrzej Duda, Rafał Trzaskowski, Włodzimierz Czarzasty, Tomasz Grodzki, Mariusz Błaszczak, Elżbieta Witek, Donald Tusk, Mateusz Morawiecki, Jarosław Kaczyński, Zbigniew Ziobro, Sławomir Mentzen, Przemysław Czarnek, Robert Biedroń, Piotr Zgorzelski, Beata Szydło, Adrian Zandberg, Krzysztof Bosak, Michał Kołodziejczak

Table S1.

*Neutral Sentences*

| Original Sentence | Translation |
| --- | --- |
| [Name] poszedł do sklepu, aby kupić produkty spożywcze. | [Name] went to the store to buy groceries. |
| Przyszedł czas, aby [Name] wybrał film na wieczór. | It was time for [Name] to choose a movie for the evening. |
| [Name] zdecydował, że spotkanie zacznie się o dziesiątej. | [Name] decided that the meeting would start at ten o'clock. |
| Ulubionym kolorem [Name] jest niebieski. | [Name]'s favorite color is blue. |
| [Name] lubi czytać książki przed snem. | [Name] likes to read books before bedtime. |
| [Name] zjadł na lunch kanapkę i wypił filiżankę kawy. | [Name] had a sandwich and a cup of coffee for lunch. |
| [Name] zawsze jeździ do pracy autobusem. | [Name] always takes the bus to work. |
| W dzień wolny [Name] lubi odwiedzać lokalne muzeum. | On a day off, [Name] likes to visit the local museum. |
| *Note:* Neutral sentences used as stimuli in the study, with their translations on the left. The politicians name were put in the place of the [NAME] placeholder. | |

Table S2.

*Political Sentences*

| Original Sentence | Translation |
| --- | --- |
| [Name] opowiedział się za bardziej rygorystycznymi przepisami dotyczącymi ochrony środowiska podczas swojej kadencji. | [Name] advocated for stricter environmental protection regulations during their term. |
| [Name] sprzeciwił się nowej ustawie o reformie podatkowej, wyrażając obawy dotyczące jej wpływu na rodziny ze średnich warstw społecznych. | [Name] opposed the new tax reform bill, expressing concerns about its impact on middle-class families. |
| Podczas debaty [Name] obiecał zwiększyć finansowanie publicznej edukacji. | During the debate, [Name] promised to increase funding for public education. |
| [Name] jest zagorzałym zwolennikiem umów o wolnym handlu. | [Name] is a staunch supporter of free trade agreements. |
| [Name] skrytykował politykę zagraniczną rządu w ostatnim przemówieniu. | [Name] criticized the government's foreign policy in the latest speech. |
| Propozycja reformy służby zdrowia przedstawiona przez [Name] spotkała się z mieszanymi reakcjami różnych interesariuszy. | The healthcare reform proposal presented by [Name] received mixed reactions from various stakeholders. |
| [Name] konsekwentnie domaga się reform wyborczych mających na celu zwiększenie uczestnictwa wyborczego. | [Name] consistently calls for electoral reforms aimed at increasing voter participation. |
| W wywiadzie [Name] wyraził sceptycyzm co do skuteczności obecnych środków cyberbezpieczeństwa. | In an interview, [Name] expressed skepticism about the effectiveness of current cybersecurity measures. |
| *Note:* Political sentences used as stimuli in the study, with their translations on the left. The politicians name were put in the place of the [NAME] placeholder. | |

**5. Statistical analysis**

| Table S3. | | | |
| --- | --- | --- | --- |
| *Model with confounds only* | | | |
|  | Dependent Variable: Valence of names | | |
|  | (1) | (2) | (3) |
| Intercept | 47.383***  (0.770) | \| 47.383*** \| \| --- \|   (0.724) | \| 47.383*** \| \| --- \|   (0.941) |
| Gender | 7.789  (4.973) |  |  |
| Trust |  | 2.155**  (0.978) |  |
| Mentions |  |  | 0.190*  (0.092) |
| Observations | 22 | 22 | 22 |
| R² | 0.109 | 0.195 | 0.175 |
| Adjusted R² | 0.065 | 0.155 | 0.134 |
| Residual Std. Error | 3.553 (df=20) | 3.615 (df=20) | 3.200 (df=20) |
| F Statistic | 2.453 (df=1; 20) | 4.852** (df=1; 20) | 4.254** (df=1; 20) |
| *Note*: *p<0.1; **p<0.05; ***p<0.01; Three models predicting the valence of politiicians’ names using the three confounders: gender of the politician (Gender), trust towards the politician (Trust), and the mean valence annotated for the tweets that included the politician (Mentions). | | | |

**6. Residuals’ QQ-plots**

Figure S3.

*QQ plots of residuals for models in Table 1*


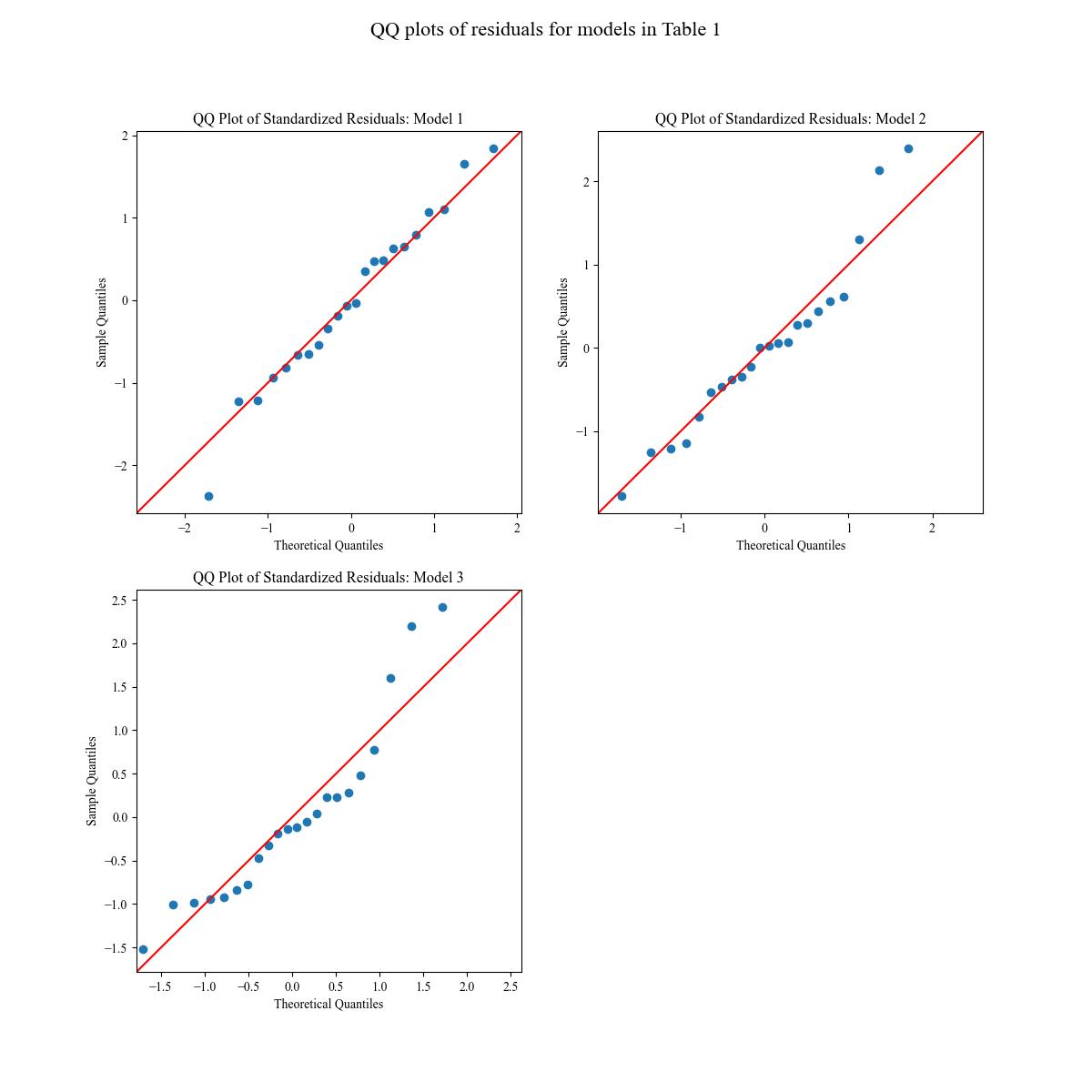


*Note***:** The QQ plot compares the sample quantiles (Y-axis) to the theoretical quantiles (X-axis) of a chosen distribution. Points close to the diagonal indicate alignment with the theoretical distribution, while deviations suggest discrepancies such as skewness or heavy tails.

Figure S4.

*QQ plots of residuals for models in Table 2*


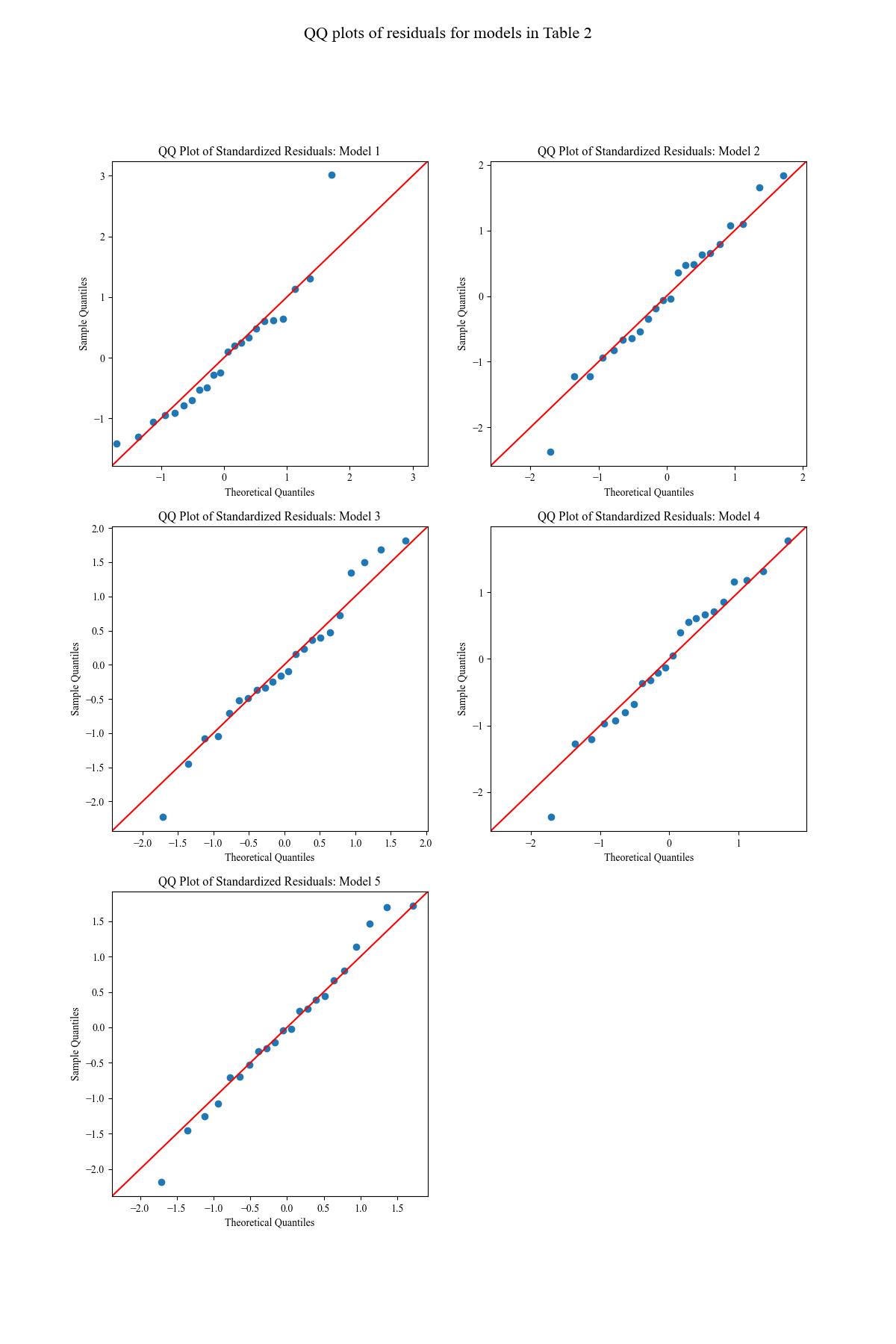


*Note***:** The QQ plot compares the sample quantiles (Y-axis) to the theoretical quantiles (X-axis) of a chosen distribution.

Figure S5.

*QQ plots of residulas for models in Table 3*


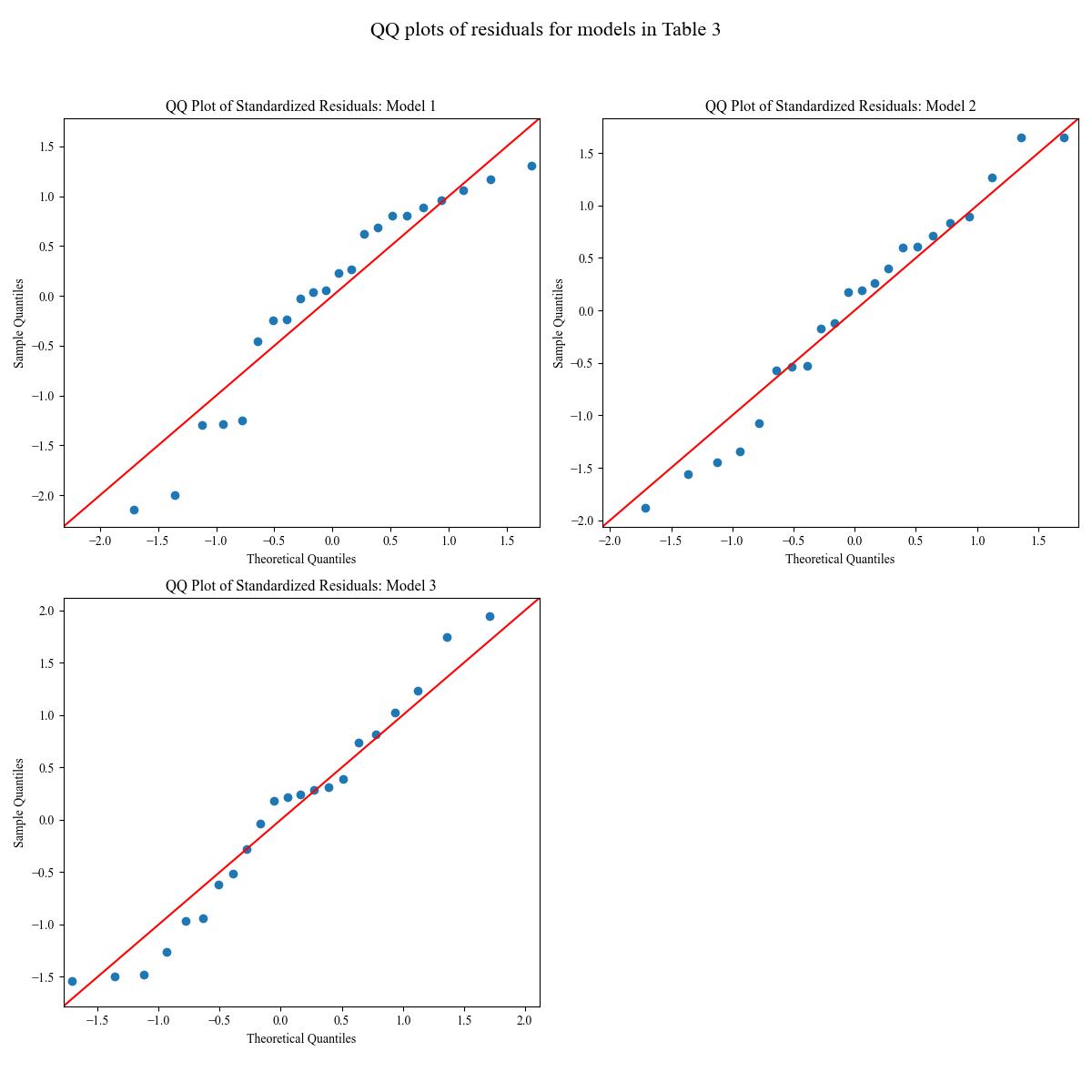


*Note***:** The QQ plot compares the sample quantiles (Y-axis) to the theoretical quantiles (X-axis) of a chosen distribution.

**6. Political Questionnaire**

**6.1 Questionnaire Administration**

To examine whether the model’s bias might be linked to the political orientations of the annotators, we administered a short post-hoc survey. Five months after the annotation process concluded, each annotator received an anonymous and voluntary questionnaire in Polish, consisting of two items from the European Social Survey [1]:

1. “The government should take action to reduce income differences.”
2. “People with a homosexual orientation, gays and lesbians, should have the freedom to arrange their lives according to their own beliefs.”

Annotators were asked to respond on a five-point scale: “Definitely agree,” “Agree,” “Neither agree nor disagree,” “Disagree,” or “Definitely disagree.” These questions were chosen to capture a wide range of possible political attitudes (economic and social) without overburdening participants. A total of 15 responses were received from the original pool of 20 annotators (75%).

**2. Questionnaire Results**

- **Item 1: “The government should take action to reduce income differences.”**
  - “Definitely agree”: 4 (26.7%)
  - “Agree”: 1 (6.7%)
  - “Neither agree nor disagree”: 5 (33.3%)
  - “Disagree”: 5 (33.3%)
  - “Definitely disagree”: 0 (0%)
- **Item 2: “People with a homosexual orientation, gays and lesbians, should have the freedom to arrange their lives according to their own beliefs.”**
  - “Definitely agree”: 13 (86.7%)
  - “Agree”: 2 (13.3%)
  - No respondents selected “Neither agree nor disagree,” “Disagree,” or “Definitely disagree.”

**3. Interpretation and Discussion**

The results suggest that our annotators hold mixed views on economic issues, with opinions about governmental intervention in income disparities ranging from strong agreement to disagreement. By contrast, responses to the social-issues item show a clear consensus in favor of progressive values.

If these political orientations were the primary source of bias in the model, one might expect significantly different sentiment scores for strongly progressive parties (e.g., Nowa Lewica) compared to more conservative parties (e.g., Konfederacja). However, our analyses consistently show lower sentiment scores for the ruling party (Zjednoczona Prawica) and higher scores for all opposition parties, including Konfederacja. This pattern could signal a broader anti-government stance, rather than one strictly driven by economic or social ideology.

Another factor to consider is that people’s personal beliefs often diverge from the official platforms of the parties they support [2]. Polish voter surveys suggest that opposition-party voters tend to share more common ground with each other than with their parties’ official positions. This might explain why the annotators—who appear strongly progressive on social issues—still assigned higher valence scores to Konfederacja members than we would predict based solely on standard left/right distinctions. Moreover, research indicates that Zjednoczona Prawica’s voters tend to be more culturally conservative yet economically left-leaning, which contrasts with our respondents’ progressive social attitudes and relatively inconsistent economic views.

Although these data do not permit precise, individual-level conclusions about how annotators’ political leanings influenced their labeling, our findings are consistent with prior work suggesting that personal political beliefs can inadvertently shape annotation outcomes [3,4,5]. The observed bias is thus likely intertwined with both general anti-government sentiment and the specific political attitudes of the annotators, underscoring the importance of careful bias monitoring in human-labeled datasets.

References

1. European Social Survey. (2020). ESS round 10 source questionnaire. ESS ERIC Headquarters c/o City, University of London.
2. CBOS. (2023a). Charakterystyka poglądów elektoratów. <https://www.cbos.pl/SPISKOM.POL/2023/K_097_23.PDF>
3. Ennser-Jedenastik, L., & Meyer, T. M. (2018). The Impact of Party Cues on Manual Coding of Political Texts. Political Science Research and Methods, 6(3), 625–633. <https://doi.org/10.1017/psrm.2017.29>
4. Van Der Velden, M. A. C. G., Reuver, M. E., Fokkens, A., Loecherbach, F., Welbers, K., & Van Atteveldt, W. (2023). Whose Truth is it Anyway? An Experiment on Annotation Bias in Times of Factual Opinion Polarization. <https://doi.org/10.31235/osf.io/nd6yr>
5. Carraro, L., Negri, P., Castelli, L., & Pastore, M. (2014). Implicit and Explicit Illusory Correlation as a Function of Political Ideology. PLoS ONE, 9(5), e96312. <https://doi.org/10.1371/journal.pone.0096312>
